# Pattern of disease recurrence and outcomes after progression of high-risk renal cell carcinoma (RCC) patients treated with adjuvant immunotherapy

**DOI:** 10.1007/s00262-026-04331-0

**Published:** 2026-02-25

**Authors:** Chiara Ciccarese, Denis Occhipinti, Daniela Arduini, Davide Di Leo, Alessio Neri, Luigi Roca, Gloria Messina, Paola Troisi, Romina Rose Pedone, Maria Antonia Fucile, Rachele Belletto, Valeria Sardaro, Chiara Ligato, Rexhina Ajdhoni, Fabiana Caliciotti, Chiara Sighinolfi, Luca Tagliaferri, Bernardo Rocco, Giampaolo Tortora, Roberto Iacovelli

**Affiliations:** 1 Medical Oncology, Department of Medical and Surgical Sciences, Fondazione Policlinico Universitario A. Gemelli IRCCS, Rome, Italy; 2 Department of Traslational Medicine and Surgery, Università Cattolica del Sacro Cuore, Rome, Italy; 3 Urology Unit, Department of Medical and Surgical Sciences, Fondazione Policlinico Universitario A. Gemelli IRCCS, Rome, Italy; 4 Department of Diagnostic Imaging and Radiation Oncology, Fondazione Policlinico Universitario A. Gemelli IRCCS, Rome, Italy; 5 Department of Radiological and Haematological Sciences, Università Cattolica del Sacro Cuore, Rome, Italy

**Keywords:** Renal cell carcinoma, Adjuvant immunotherapy, Disease recurrence, Oligometastatic disease, Loco-regional therapy, Multidisciplinary management

## Abstract

**Background:**

Radical or partial nephrectomy followed by adjuvant pembrolizumab is the standard of care for high-risk localized renal cell carcinoma (RCC), yet around 40% of patients relapse within 5 years. We investigated patterns of disease recurrence and the clinical management of RCC patients treated with adjuvant immunotherapy.

**Materials and methods:**

We collected patients with high-risk RCC who received adjuvant immunotherapy after radical surgery in our Institution. The primary endpoint was the rate and pattern of disease recurrence. Secondary endpoints were disease-free survival (DFS), overall survival (OS), post-progression survival (OS2) and treatments at recurrence.

**Results:**

From March 2018 to September 2025, 70 patients were included, most received adjuvant pembrolizumab (71%), followed by nivolumab + ipilimumab (16%), and nivolumab monotherapy (13%). 15 patients (21%) experienced recurrence, including 7 (10%) who relapsed on adjuvant treatment. Oligometastatic disease was observed in 10 cases (67%), mainly involving lung (60%), lymph nodes (33%) and renal bed (13%). At recurrence, 9 patients (60%) started first-line therapy, while 5 patients (33%) received loco-regional treatments. After a median follow-up of 30.2 months, 30-month DFS and OS rates were 74% and 94%, respectively, in the overall population. Among patients who progressed, the 24-month OS2 rate was 100% after local therapy alone and 86% with systemic therapy.

**Conclusions:**

High-risk RCC patients treated with adjuvant immunotherapy remain at considerable risk of relapse, frequently with oligometastatic disease. Excellent post-progression outcomes after loco-regional treatment support a multidisciplinary, metastasis-directed approach to recurrence after adjuvant immunotherapy.

## Introduction

Renal cell carcinoma (RCC) accounts for approximately 3% of all cancers, with the highest incidence observed in Western Countries, 80.980 and more than 430.000 new cases expected to be diagnosed in 2025 in the United States and globally, respectively [1, 2]. Surgery with curative intent remains the standard of care for patients with localized (stages I-III) RCC. However, about 35–50% of patients with high-risk features will experience disease recurrence [3, 4]. To date, a univocal classification that estimates the risk of relapse for localized RCC still lacks; the various risk models (i.e. UISS, SSIGN, Leibovich, Karakiewicz) are based only on clinical-pathological features and have a limited applicability in clinical practice, other than guiding the timing of follow-up [3]. Many efforts were made in the past, trying to reduce the risk of tumor recurrence with adjuvant VEGFR tyrosine kinase inhibitors (VEGFR-TKIs), with no meaningful results in terms of both statistical and clinical significant advantage over standard observation. The only formally positive trial (S-TRAC) that led to FDA approval of adjuvant sunitinib for 1 year in high-risk clear cell RCC patients, demonstrated a 24% reduction in the risk of disease recurrence, which did not translate in an overall survival (OS) benefit at the cost of relevant toxicity [5]. Recently, immunotherapy with immune checkpoint inhibitors (ICIs) directed against PD1/PD-L1 has revolutionized the therapeutic landscape of kidney cancer, with a key role also in the adjuvant setting. Based on the latest results of KEYNOTE-564 trial, adjuvant pembrolizumab for 1 year compared to placebo is the first ICI to significantly improve disease-free survival (DFS) and OS in high-risk RCC patients (pT2 grade 4 or worse), with a 28% reduction in the risk of recurrence (HR 0.72, 95% CI 0.59–0.87) and 38% reduction in the risk of death (HR 0.62; 95% CI 0.44–0.87), respectively [6, 7]. Consequently, adjuvant pembrolizumab is now the standard treatment for patients with high-risk localized RCC following radical or partial nephrectomy. More recently the RAMPART trial reported a significant improvement in DFS for the combination of durvalumab plus tremelimumab over placebo (3 year DFS rate 81% vs. 73%; HR 0.65, 95% CI 0.45–0.93, *p* < 0.01) in high-risk RCC patients according to Leibovich score, while the data for OS are still immature [8]. Despite this evidence, approximately 30–40% of patients will eventually relapse within five years, rising questions about the appropriate selection of patients who actually benefit from adjuvant pembrolizumab, but also about the appropriate management of disease progression post-adjuvant therapy.

Despite adjuvant immunotherapy, RCC remains a disease with a high risk of recurrence, highlighting the need for improved therapeutic strategies in the adjuvant setting. In this study, we described a retrospective cohort of localized RCC patients treated with adjuvant ICIs, the pattern of disease recurrence and the treatment received in case of disease relapse.

## Materials and methods

### Patients

In this analysis, we included consecutive patients with high-risk RCC who received adjuvant ICIs after radical surgery in our Institution. Baseline characteristics required for the inclusion in the analysis were age, tumor histology, tumor stage and grading, presence of sarcomatoid component, and prognostic risk classification according to KEYNOTE-564 definition. Among the selected patients, only those with complete baseline characteristics at the time of initiating adjuvant therapy were included in the final analysis.

The primary endpoint was to assess the rate and pattern of disease recurrence after adjuvant ICI. Secondary endpoints included patient’ outcomes in terms of DFS, OS and OS2 as well as the type of treatment received including local procedures and medical therapy in case of tumor progression.

Subgroup analyses were performed to assess the impact of the type of adjuvant therapy (in particular the role of pembrolizumab) in terms of efficacy (i.e. DFS and OS).

### Statistics

Baseline values were expressed as the median value. Baseline was defined as the start date of adjuvant ICI. DFS was evaluated from the baseline to disease recurrence or death. OS was evaluated from the baseline (start of adjuvant ICI) to death or the last follow-up; OS2 was evaluated from disease progression during or after adjuvant ICI to death or the last follow-up. All survivals were estimated using the Kaplan–Meier method and compared across groups using the log-rank test. A Chi square or t-test was used to compare groups when appropriate. All the variables were considered to be significant if *p* < 0.05. The PASW software (Predictive Analytics SoftWare; v 21; IBM SPSS) was used for the analysis.

## Results

### Study population

From March 2018 to September 2025 a total of 70 patients met criteria to be included in the final analysis.

50 out of 70 patients (71%) received pembrolizumab as adjuvant therapy; 11 patients (16%) were treated with the combination of nivolumab plus ipilimumab; and the remaining 9 patients (13%) received nivolumab monotherapy. At time of immunotherapy initiation, KN-564 risk groups were 84% (n = 59) intermediate-high, 6% (n = 4) high, and 10% (n = 7) of patients started adjuvant immunotherapy after radical metastasectomy (M1NED).

At the time of the analysis, 47% of patients (n = 33) completed the adjuvant therapy (one year for pembrolizumab, 6 months for either the combination of nivolumab plus ipilimumab and nivolumab monotherapy); 20% (n = 14) and 10% (n = 7) discontinued treatment because of toxicity and disease progression, respectively; and 23% (n = 16) were still on treatment. Baseline characteristics of the included patients are reported in the Table 1.

**Table 1:** Baseline characteristics of the patients

Baseline characteristics	Patients (*N* = 70)
Median age (range)	63.5 (34.7 – 83.6)
Male sex, *n* (%)	48 (69)
Primary tumour stage, *n* (%)	
T1	5 (7)
T2	5 (7)
T3	58 (83)
T4	2 (3)
Tumour nuclear grade, *n* (%)	
Grade 1	1 (1)
Grade 2	16 (23)
Grade 3	27 (39)
Grade 4	21 (30)
Lymph node stage, *n* (%)	
N0	66 (94)
N1	4 (6)
Metastatic stage, *n* (%)	
M0	62 (89)
M1NED	8 (11)
Clear cell histology, *n* (%)	67 (96)
Presence of sarcomatoid features, *n* (%)	16 (23)
Risk category according to KN-564, *n* (%)	
Intermediate-high	58 (83)
High	4 (6)
M1NED	8 (11)
Type of adjuvant immunotherapy, *n* (%)	
Pembrolizumab	50 (71)
Nivolumab + ipilimumab	11 (16)
Nivolumab	9 (13)

Adj: adjuvant; Pt: patient; M1 NED: patients who started adjuvant immunotherapy after radical metastasectomy

### Disease recurrence

After a median follow-up of 30.2 months (26.8–33.6 months), 8 patients (11%) had disease recurrence after the end of adjuvant immunotherapy and 7 (10%) during adjuvant immunotherapy. In 9 of these cases (60%) the number of metastases was 1 and another patient had up to 3 sites. The most frequent sites included lung (60%, *n* = 9), lymph node (33%, *n* = 5), and renal bed (13%, *n* = 2).

**Fig. 1:**
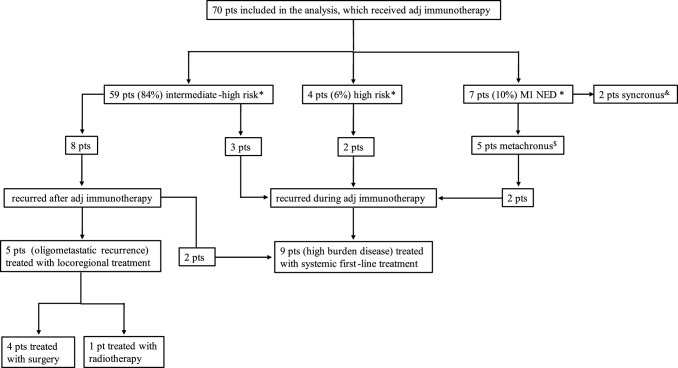
Treatment flow

The management of disease recurrence included the start of a systemic first-line therapy in 9 patients (60%), while 5 patients (33%) received a loco-regional treatment (1 patient radiotherapy and 4 patients radical metastasectomy) (Fig. 1); all these patients were alive without subsequent disease progression. One patient progressed during adjuvant pembrolizumab and died without receiving any subsequent treatments. Among the 9 patients treated with systemic therapy, all of them received a VEGFR-TKI-based treatment, either as monotherapy (56%, *n* = 5) or associated with an ICI (44%, *n* = 4). Post-adjuvant treatments of the included patients are reported in the Table 2.

**Table 2:** Therapeutic strategy of post-adjuvant disease recurrence

Post-adjuvant therapy	*n* (%)
None	56 (80)
Loco-regional treatment	5 (7)
Surgery	4 (6)
Radiotherapy	1 (1)
Systemic therapy	9 (13)
VEGFR-TKI	4 (6)
ICI + ICI (nivolumab + ipilimumab)	0 (0)
VEGFR-TKI + ICI	4 (6)
Other	1 (1)^#^

^#^lenvatinib + belzutifan ICI = immune checkpoint inhibitor; TKI = tyrosine kinase inhibitor

### Efficacy outcomes

In the overall population, the rate of patients free from disease recurrence at 30 months was 74% and the 30 months OS rate was 94%.

In the subgroup of patients treated with adjuvant pembrolizumab, the 30 months DFS rate was 72% and the 30 months OS rate was 92%.

For patients with a DFS event, after a median follow-up of 24.5 months, the 24 months OS2 rate was 100% for patients who received a local treatment and 86% for patients who received systemic treatment.

## Discussion

In this study, we retrospectively evaluated a single-center cohort of 70 patients with high-risk localized RCC who received adjuvant immunotherapy after radical surgery, with the aim of analyzing the rate, features, and clinical management of disease recurrence. To our knowledge, no prospective data are currently available evaluating the typical patterns of failure and the optimal therapeutic approaches in this critical novel setting.

Approximately 35% of patients experience disease progression within four years despite adjuvant immunotherapy [6]. Our findings align with these results, with a 30 month DFS rate of 74% in the overall population and 72% for patients treated with adjuvant pembrolizumab. It is noteworthy that our cohort mirrored that of the KN-564 study, with a clear preponderance (over 80%) of intermediate-high risk patients.

Commonly, lung is the most common site of solitary metastases (about 17%), followed by lymph nodes (12%) and bone (11%) [9]. In our experience, about two third of patients with recurrence had ≤ 3 metastases, and we did confirm lung (more than half of the cases) as the most frequent site of disease recurrence, followed by lymph nodes and renal bed.

Actually, early use of ICIs in the adjuvant setting has created a need to clarify the appropriate management of metastatic disease after failure of adjuvant immunotherapy. Some key factors are important to consider, including the time to disease recurrence after adjuvant therapy (i.e. early versus delayed—even with no consensus about the exact cutoff), the disease burden, the biology of the tumor. Certainly, the disease burden (which accounts for the number and the volume of metastatic lesions) and the site of metastases are crucial for guiding the subsequent treatment algorithm. Oligo-metastatic disease, defined as a limited metastatic burden typically ranging from one to five distant metastases, is generally associated with better prognosis and may benefit from loco-regional therapeutic approaches [10]. Oligo-metastatic RCC patients (with ≤ 3 metastases) have longer OS compared to poly-metastatic ones, thus leaving more space for a less aggressive therapeutic approach [11]. In the specific subgroup of oligo-recurrent disease, metastasis-directed treatments (MDT), such as stereotactic body radiation therapy (SBRT) and metastasectomy, have demonstrated efficacy in achieving disease control, delaying the need for systemic treatment and preserving patients’ quality of life (QoL), without impairing survival outcomes [12–17]. Indeed, even though we have no data in the post-adjuvant immunotherapy setting, SBRT can be considered a valid alternative to systemic treatment in oligo-metastatic RCC, achieving a disease control rate of 90% [14, 15]. Similarly, radical metastasectomy remains a viable option for selected patients, potentially leading to prolonged disease control, without requiring systemic treatment [17, 18]. Results of our analysis are in line with these findings reporting a 24 months OS2 rate of 100% in patients treated with loco-regional therapies.

**Fig. 2:**
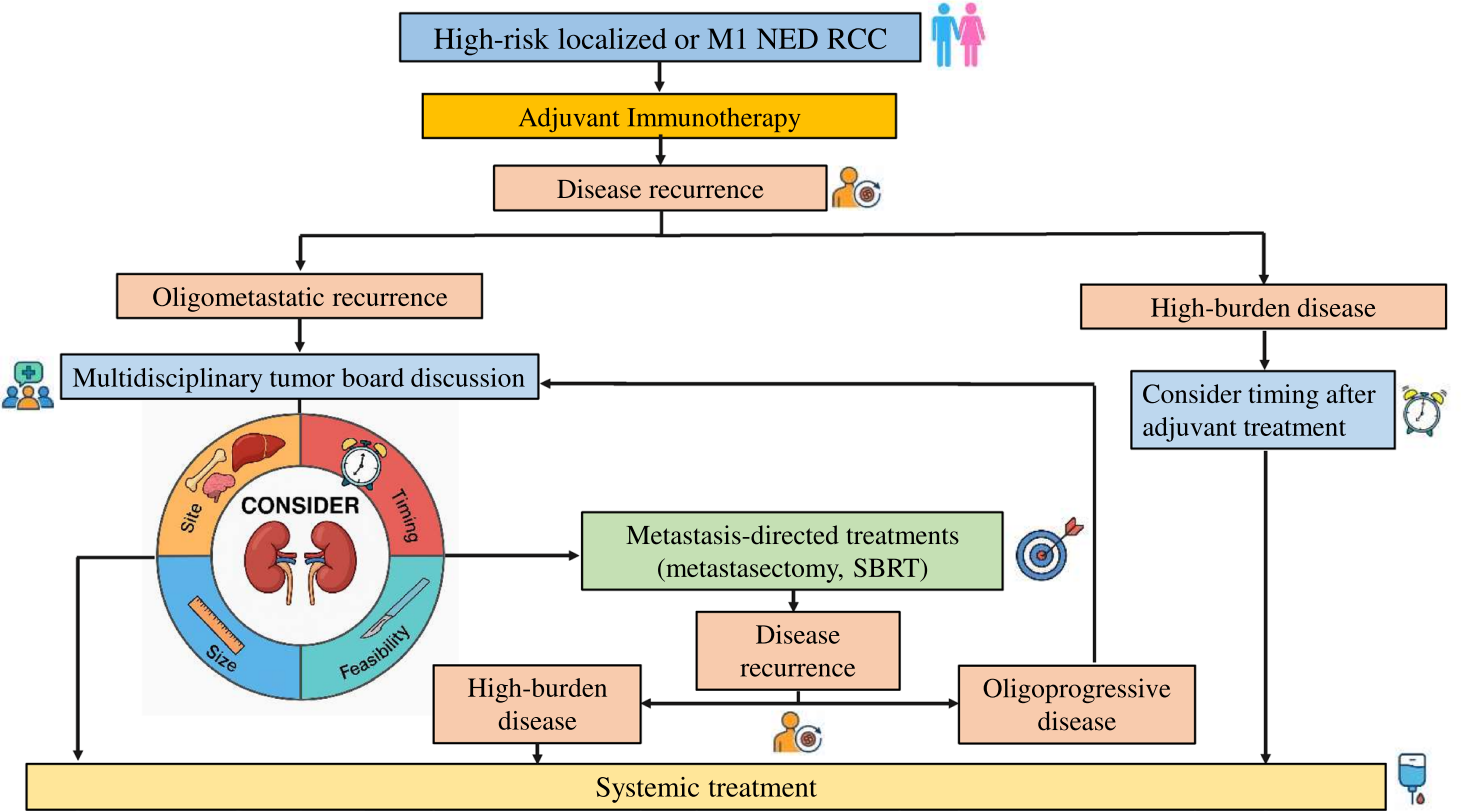
Suggested management of disease recurrence after adjuvant immunotherapy

Conversely, poly-metastatic patients with a higher disease burden are more likely to benefit from systemic treatments. In our Institution, more than half of the patients (60%) started systemic first-line therapy after disease recurrence. Notably, all the patients recurred during adjuvant immunotherapy had a high burden disease and were treated with systemic therapy, underlining a more aggressive biology. There is a wide consensus in considering that treatment should in any case include a therapy based on VEGFR-TKIs, while the role of re-challenge with ICIs is not clear after adjuvant immunotherapy failure. Two recent phase 3 trials, CONTACT-03 and TiNivo-2, although designed in the metastatic setting, failed to demonstrate the efficacy of immunotherapy re-challenge in combination with a VEGFR-TKI compared to a VEGFR-TKI monotherapy [19, 20]. Therefore, while waiting for prospective evidence focused on this setting, re-proposing an immunotherapy-based treatment, especially in particular circumstances (disease recurrence during adjuvant therapy or shortly after the end; multi-district recurrence with symptomatic/bulky disease and in sites associated with worse prognosis), does not seem to be the therapeutic strategy recommended at the moment [21]. Recent real-world evidence has provided additional insight into the management of recurrent RCC after adjuvant immunotherapy [22]. In this multicenter analysis, patients relapsing after adjuvant ICI derived meaningful benefit from subsequent systemic therapies. In particular, favorable-risk patients seemed to benefit more from VEGFR-TKI than from further IO-based combinations, highlighting the possible predominance of angiogenic rather than immunogenic pathways in tumors recurring after adjuvant immunotherapy. Our data are consistent with these findings, supporting a personalized approach in which treatment selection after adjuvant immunotherapy should consider both timing and pattern of recurrence, as well as underlying risk and biological profile (Fig. 2).

These data underscore the need for improved patients selection, discover predictive biomarkers, and develop even more effective therapeutic strategies in the adjuvant setting [23, 24]. Ongoing clinical trials are assessing the efficacy of different immunotherapy-based combinations to enhance outcomes of radically resected high-risk localized RCC patients, including LITESPARK-022 phase 3 trial (NCT05239728) with pembrolizumab plus belzutifan (HIF-2*α* inhibitor), INTerpath-004 study (NCT06307431) with pembrolizumab plus an individualized neoantigen therapy mRNA-4157 (V940).

Several limitations can impair the results of our paper. The retrospective nature of our data, and the consequent selection bias, represents the major limitation of our study; moreover, the small sample size, together with the heterogeneity of the type and duration of ICIs received as adjuvant treatment, suggest additional prospective studies to validate our findings. Finally, a longer follow-up will add help in addressing the optimal algorithm in case of post-adjuvant disease recurrence.

In conclusion, our study confirms that, given the elevated risk of recurrence of high-risk RCC treated with adjuvant immunotherapy, the optimal treatment algorithm of these patients in the post-adjuvant setting remains one of the most important challenges to be addressed. A multidisciplinary approach that involve highly specialized clinicians, is crucial for guarantee a proper patients’ management.

## Data Availability

The datasets generated and/or analysed during the current study contain sensitive clinical information and are not publicly available due to privacy and ethical restrictions. De-identified data may be made available from the corresponding author on reasonable request and subject to approval by the institutional Ethics Committee.

## Abbreviations

RCC: Renal cell carcinoma
ICI: Immune checkpoint inhibitor
DFS: Disease-free survival
OS: Overall survival
OS2: Overall survival after disease progression (post-progression survival)
VEGFR-TKI: Vascular endothelial growth factor receptor tyrosine kinase inhibitor
SBRT: Stereotactic body radiation therapy
MDT: Metastasis-directed therapy
KIM-1: Kidney injury molecule-1, a transmembrane glycoprotein and potential biomarker in RCC
